# Puckering vs. Localisation: Contrasting Nanoscale Lithography and Wear Mechanisms in MoS_2_ and Graphene on SiO_2_

**DOI:** 10.3390/ma19091738

**Published:** 2026-04-24

**Authors:** Miljan Dašić, Igor Stanković

**Affiliations:** 1 Institute of Physics Belgrade, University of Belgrade, Pregrevica 118, 11080 Zemun, Serbia; 2 Departamento de Ingenería Mecánica, Universidad Técnica Federico Santa María, Av. España 1680, Valparaíso 2390123, Chile; igor.stankovic@usm.cl

**Keywords:** nanotribology, friction, wear, all-atom molecular dynamics, 2D materials

## Abstract

Two-dimensional (2D) materials are promising candidates for nanoscale wear-protective coatings. The mechanisms governing their tribological behaviour (i.e., friction and wear) are material-dependent. In this work, we use atomistic molecular dynamics simulations to investigate nanoscale sliding, friction, and lithographic tracks in two 2D materials, graphene and MoS_2_, both placed on a SiO_2_ substrate. Our results reveal fundamentally different deformation mechanisms in the two materials, where deformation comes as a consequence of applied normal load. MoS_2_ deforms via the formation of a stable out-of-plane pucker beneath the contact, enabling efficient absorption and elastic redistribution of mechanical energy within the coating as well as simultaneous reduction of plastic deformation of the underlying material. Wear prevention in the substrate comes at the cost of localised damage to the MoS_2_ layer along the sliding path once it reaches the rupture point. On the contrary, graphene exhibits strongly localised deformation due to its high in-plane stiffness and atomic thickness, leading to plastic deformation of the underlying material and mitigating layer damage. These findings provide clear design guidelines for 2D coatings in nanotribological applications, and highlight layered materials, such as MoS_2_, as particularly effective for wear protection.

## 1. Introduction

Reducing friction and wear between sliding interfaces in mechanical systems remains a significant challenge across all length scales, due to the complex interplay of contact mechanics, material properties, and surface phenomena—from atomistic to macroscopic levels [1,2,3]. The need for low friction and anti-wear performance of materials becomes particularly important when characteristic dimensions approach the micro- and nanoscale, where contact effects and surface forces dominate tribological behaviour [4,5]. At these length scales, conventional liquid lubricants often become ineffective due to the confinement effects, volatility, chemical instability, and similar factors [6,7,8]. Such shortcomings have sparked significant research interest in robust, atomically thin protective coatings that can withstand high contact stresses while maintaining low friction. In this context, two-dimensional (2D) materials, especially graphene [9,10,11,12] and molybdenum disulfide (MoS_2_) [13,14,15,16,17], have emerged as promising candidates for wear protection and solid lubrication at the nanoscale.

Graphene, a single atomic layer of $sp^{2}$-bonded carbon atoms arranged in a hexagonal lattice, combines exceptional in-plane stiffness and strength with low interlayer shear resistance in multilayer assemblies [18,19,20]. These nanomechanical properties distinguish graphene as an attractive ultra-thin protective coating for reducing friction and mitigating wear. Atomic Force Microscopy (AFM) experimental studies have shown that graphene exhibits distinct wear regimes as a function of the applied normal load [21,22]. At lower loads, graphene undergoes plastic deformation, but remains continuous. On the other hand, at higher loads, graphene fractures, tears, and eventually detaches from the substrate, consequently exposing the underlying substrate material. Importantly, the tribological performance of graphene strongly depends on its thickness: while single- and bilayer graphene can enhance the load-carrying capacity of the substrate, effective wear protection is achieved only with multilayer graphene films with a thickness of several nanometers [23]. Beyond its role as a passive protective layer, graphene also exhibits remarkable resistance to localised mechanical manipulations. AFM experiments on dynamic ploughing and scratching have revealed that graphene can withstand substantial local stresses before rupture, with deformation and cutting processes governed by the applied force and tip–sample interaction [24,25]. These studies provide valuable insights into the nanoscale mechanical response of graphene, including strain accumulation, fracture initiation, and material removal mechanisms, which are directly relevant to understanding wear in graphene under nanoscale sliding [23,24,25].

Molybdenum disulfide (MoS_2_) is another prominent 2D material, recognised also for its superior solid lubrication properties [26,27,28]. Its lamellar crystal structure, composed of weakly interacting S–Mo–S layers, facilitates interlayer sliding and results in low shear strength [29,30]. Analogous to graphene, MoS_2_ can act as a sacrificial layer that accommodates shear, thereby protecting the underlying substrate from damage. While graphene offers exceptional mechanical strength and chemical inertness, MoS_2_ provides intrinsically low shear resistance, making the comparison between these two materials particularly relevant for nanoscale wear protection strategies.

A detailed understanding of the wear mechanisms, thickness dependence, and failure modes of graphene and MoS_2_ at the nanoscale is therefore essential for the study of nanotribology of 2D layered materials. Such knowledge enables the rational design of advanced protective coatings and solid lubricants for micro- and nano-electromechanical systems (MEMSs and NEMSs) [31,32,33,34] and also allows understanding the mechanical behaviour of more complex systems involving 2D materials with adsorbed or intercalated small molecules [35,36].

## 2. Materials and Methods

All simulations presented in this work use the classical molecular dynamics (MD) method as implemented in the Large-scale Atomic/Molecular Massively Parallel Simulator (LAMMPS) [37,38]. In the system description, each atomic type is defined by its Lennard-Jones (LJ) parameters and charge. To capture all the relevant phenomena, we chose the all-atom level of description of materials (i.e., All-Atom MD simulations) [36,39,40,41,42,43,44,45]. The MD simulation setup reproduces nanoscale deformation, friction, and wear processes in 2D materials on oxide substrates, similar to conditions in AFM nano-lithography and friction force manipulation experiments [24,46,47,48]. We fully leveraged parallel simulation by running a batch submission script suitable for High-Performance Computing (HPC) environments. A velocity–Verlet algorithm with a time step of 0.5 fs allowed numerical stability of the equations of motion in high-load contact during sliding.

In the following, we provide an overview of pair- and many-body-bond-order potentials used to describe covalently bonded and layered materials. Tersoff-type potential parametrised for silicon–oxygen systems enabled realistic modelling of elastic deformation, plastic rearrangements, and bond rupture within the oxide under high-contact stresses [49,50]. Carbon–carbon (C–C) interactions in graphene were modelled using a Tersoff-type potential, which enables an accurate description of bond stretching, angular interactions, and bond breaking under mechanical loading and wear [49,50]. The adhesion between SiO_2_ and graphene was described by a van der Waals interaction potential. The LJ parameters (σ,ε) assigned to Si, O, and C atoms were (3.8264 Å, 0.01301 eV), (3.1181 Å, 0.00650 eV), and (3.4121 Å, 0.00239 eV), respectively [24]. MoS_2_ was modelled using a reactive bond-order potential specifically developed for transition metal dichalcogenides [51]. This potential captures directional Mo–S bonding, angular interactions, and interlayer shear, which are essential for describing frictional sliding and wear in layered MoS_2_ systems. For MoS_2_ adhesion, Mo atoms were described by σ=4.43 Å, ε=0.116kcal/mol, and charge +0.5e, while S atoms were assigned σ=3.34 Å, ε=0.4983kcal/mol, and charge −0.25e [52]. The LJ parameters defining the pair interaction of two atoms belonging to the same type (labelled as α) are ($\epsilon_{\alpha \alpha }$, $\sigma_{\alpha \alpha }$), while all the atoms belonging to the same type have the same charge $q_{\alpha }$. Cross-interaction parameters between different atomic types are calculated using the Lorentz–Berthelot mixing rules [53], i.e., $\epsilon_{\alpha \beta }=\sqrt{\epsilon_{\alpha \alpha }\epsilon_{\beta \beta }}$, $\sigma_{\alpha \beta }=\frac{\sigma_{\alpha \alpha }+\sigma_{\beta \beta }}{2}$. All interatomic interactions were treated consistently within the chosen potential framework, allowing energy dissipation, structural rearrangements, and material removal during sliding.

**Figure 1:**
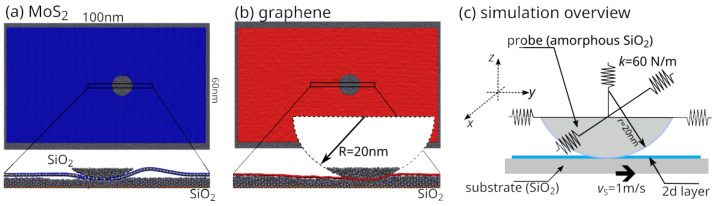
Configuration snapshots of atomistic models used in MD simulations: (**a**) MoS_2_ on SiO_2_ substrate and (**b**) graphene on SiO_2_ substrate. Simulation cell dimensions are approximately: 100 nm × 60 nm. The rigid spherical SiO_2_ indenter (representing the tip in AFM experiments) with an effective radius of R=20 nm is sliding over the substrate while normal load is simultaneously applied. (**c**) Schematic representation of the implemented simulation setup, which is designed following the experimental AFM setup shown in panels (**a**,**b**) of this figure. The values of the parameters defining the simulation setup are sliding velocity $v_{S}=1$ m/s and spring stiffness coefficient k=60 N/m, which is the same for the elastic springs attached to the probe in all three Cartesian directions.

The simulated systems correspond to the geometries illustrated schematically in Figure 1. Two distinct configurations were considered, namely: (a) MoS_2_ sheet on SiO_2_ substrate and (b) graphene sheet on SiO_2_ substrate. The simulation cell dimensions were approximately: 100 nm in the sliding direction and 60 nm in the transverse in-plane direction. Periodic boundary conditions (PBCs) were applied only to the SiO_2_ substrate in the in-plane directions, while the 2D materials (graphene and MoS_2_) were modeled as finite flakes. This choice was made to avoid artificial interactions between deformation fields and their periodic images. In particular, in the case of MoS_2_, the formation of an out-of-plane pucker under the indenter would interact with its periodic replicas if fully periodic boundary conditions were imposed on the 2D layer, leading to non-physical constraints on deformation and stress redistribution.

The SiO_2_ substrate was modelled as a slab. Its bottom layers were connected to so-called *ghost atoms* acting as fixed centres around which atoms can move, via an 8 kN/m spring to suppress deformation under AFM tip pressure and act as a rigid bulk. In contrast, the upper layers were fully dynamic and allowed to deform in response to applied loads. We placed a single layer of MoS_2_ or graphene on top of the SiO_2_ substrate.

Mechanical loading was applied using a rigid spherical SiO_2_ indenter representing a nanoscale contact, with an effective radius of R=20 nm (check Figure 1). The indenter probe was positioned above the centre of the simulation cell. Such a geometry enabled a controlled investigation of stress distribution, interfacial shear, and wear processes in the 2D coatings. The graphene was studied at normal loads of 0.75 μN and 0.5 μN, while MoS_2_ was studied at normal loads of 0.65 μN and 0.5 μN. For our setup, 0.65 μN was the maximum load that MoS_2_ could sustain without immediately breaking. The 0.5 µN load was selected as a common reference condition for direct comparison between the two materials. The higher loads correspond to material-specific regimes approaching the onset of structural failure, and are therefore used to analyse rupture behaviour rather than for direct comparison.

The probe is connected to the support via harmonic elastic springs in all three orthogonal directions (*x*-, *y*-, and *z*-) to measure the lateral and normal forces, similar to a Friction Force Microscopy (FFM) experiment. The probe has a spring stiffness of k=60 N/m in all three orthogonal directions, cf. Figure 1c. The probe support is fixed while the SiO_2_ substrate (i.e., the ghost atoms) moves at a sliding velocity $v_{S}=1$ m/s (similar to AFM experiment). Normal loading was applied by displacing the rigid spherical indenter’s support vertically toward the coating on the substrate until the desired normal contact force was achieved. Subsequently, lateral sliding was imposed by prescribing a constant sliding velocity $v_{S}=1$ m/s to the ghost atoms along the in-plane direction, mimicking AFM-based scratching and wear experiments. The same “loading-plus-sliding” protocol was applied to both graphene and MoS_2_ systems to enable direct comparison of their deformation and wear behaviour under comparable mechanical conditions, with a direct comparison performed at a common normal load of 0.5 µN. Before sliding, the systems were relaxed by thermal equilibration at T=300 K to remove residual stresses. Temperature control was applied via a Nose–Hoover thermostat.

Atomic trajectories, forces, and energies were recorded during the MD simulations. Structural analysis focused on atomic displacements, coordination changes, bond rupture events, and local deformations of both 2D (MoS_2_/graphene) layers and SiO_2_ substrate. These analyses enabled the identification of distinct deformation and failure regimes, including elastic and plastic deformation, the onset of fracture, material removal, and exposure of the underlying substrate. Atomistic mechanisms observed in the simulations were instrumental in interpreting the differences in wear resistance and load-bearing behaviour between graphene and MoS_2_ coatings.

## 3. Results

**Figure 2:**
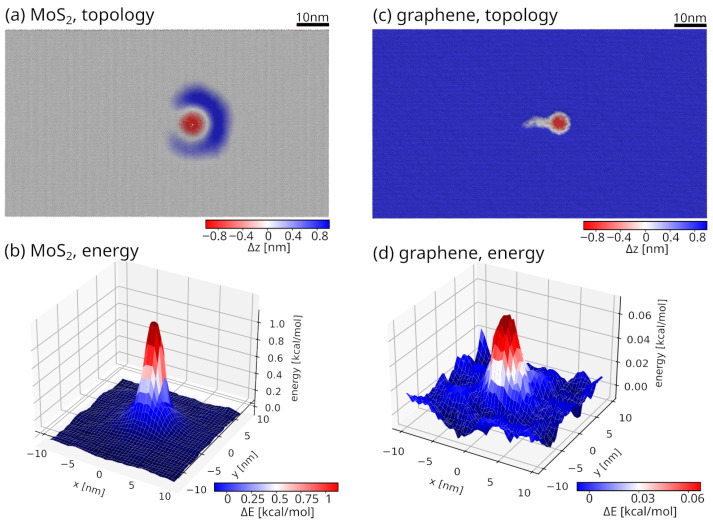
Spatial maps of vertical atomic displacements Δz and per-atom potential energy in the vicinity of the indenter in the 2D layer induced by nanomechanical contact for: MoS_2_ and graphene on SiO_2_ substrate. Colour maps show the out-of-plane (i.e., along the *z*-direction) displacement field, with the scale indicated by the colour bars. MoS_2_ is a plane in the midpoint between forced down (red) and puckered material (blue). On the other hand, graphene does not form puckers, and blue atoms correspond to undeformed graphene. The per-atom energy localised in MoS_2_ is nearly twenty times that in graphene, indicating substantially stronger energy absorption and deformation within the MoS_2_ layer. The scale bar corresponds to 10 nm, as indicated in this figure.

We have quantified the deformation of both MoS_2_ and graphene on SiO_2_ substrate under controlled and similar nanomechanical conditions, the results of which are shown in Figure 2. In the case of MoS_2_, cf. Figure 2a, the displacement results in the pronounced puckering beneath the contact with the indenter. This pucker extends laterally beyond the immediate region of contact with the tip, reflecting the layered MoS_2_ structure’s ability to accommodate mechanical load through out-of-plane bending and interlayer shear. The result presented in Figure 2b shows a strong localisation of the per-atom potential energy within the pucker region, with peak energy values nearly twenty times higher than those observed in graphene (shown in Figure 2d). This result demonstrates that MoS_2_ absorbs a significant fraction (i.e., 95%) of the applied mechanical energy through internal deformation, thereby absorbing the tip-induced disturbance and shielding the underlying SiO_2_ substrate from excessive stress.

In contrast, graphene, see Figure 2c, exhibits a much more localised deformation without extended puckering. The displacement field is confined to a narrow region around the contact point with the tip, and the per-atom energy accumulation remains comparatively low. Owing to its high in-plane stiffness and atomic thickness, graphene resists bending and stores less deformation energy, resulting in a more concentrated stress distribution at the interface. The in-plane stiffness (two-dimensional Young’s modulus, *E*) of monolayer graphene has been experimentally measured to be approximately 340 N/m using AFM nanoindentation techniques [54]. For monolayer MoS_2_, reported experimental value is 180 N/m [55]. For a comparison with three-dimensional (3D) materials, an effective Young’s modulus *E* can be obtained by dividing the 2D Young’s modulus by an effective layer thickness *t*. Using t=0.335nm for graphene yields E≈1.0TPa=1000GPa, while adopting t=0.65nm for MoS_2_ gives E≈200–280GPa.

These observations highlight fundamentally different mechanisms for load accommodation in the two studied materials. MoS_2_ protects the substrate by puckering, absorbing, and redistributing deformation energy, whereas graphene responds primarily through localised elastic deflection, with limited energy storage within the coating.

**Figure 3:**
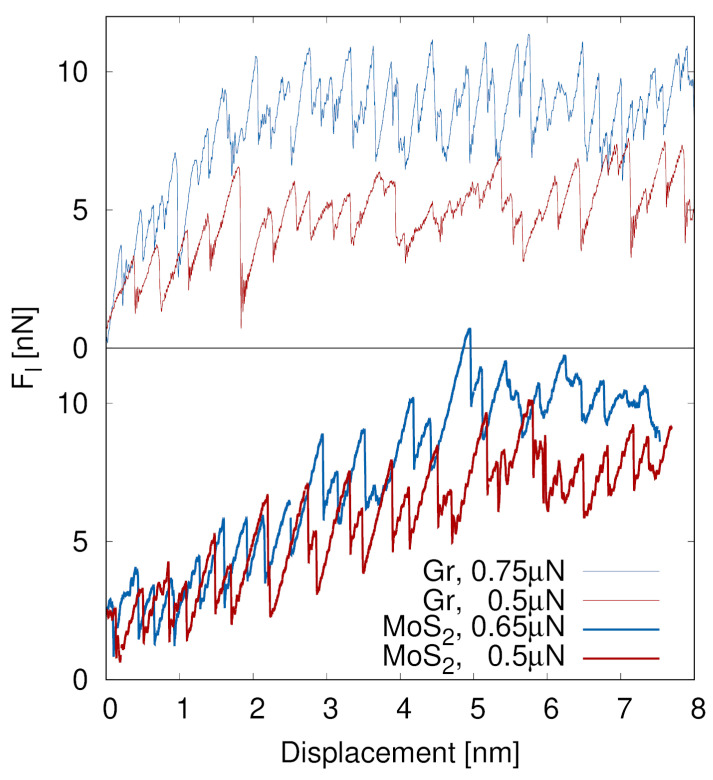
Lateral force $F_{l}$ as a function of sliding displacement for graphene (Gr) and MoS_2_ coatings under different normal loads. Upper panel: graphene at normal loads of 0.75 μN and 0.5 μN. Lower panel: MoS_2_ at normal loads of 0.65 μN and 0.5 μN. For our setup, 0.65 μN was the maximum load that MoS_2_ could sustain without immediately breaking. The sawtooth-like response of lateral force reflects the stick–slip motion during sliding. Since both graphene and molybdenum-disulfide have a crystal structure, observation of stick–slip friction is expected.

In the following, a direct comparison between graphene and MoS_2_ is performed at the common normal load of 0.5 µN. The higher-load cases are analysed separately to investigate material-specific responses near the onset of rupture in MoS_2_ and to observe a difference in graphene under sufficiently high loads. Figure 3 presents the lateral force $F_{l}$ as a function of sliding displacement for graphene and MoS_2_ coatings under different normal loads. In all cases, the lateral force exhibits a pronounced sawtooth-like behaviour characteristic of stick–slip motion at the scale of lattice constant (i.e., approximately 0.3 nm), reflecting the repeated cycles of pinning and consequent elastic loading of the probe spring, followed by sudden slip events at the 2D material interface.

On slightly larger time and displacement scales, the graphene lateral force (shown in the upper panel of Figure 3) increases rapidly during the initial stage of sliding (up to 2 nm displacement) and reaches relatively high average values, particularly at the higher normal load of 0.75 μN. The initial upward trend in the mean value of $F_{l}$ suggests progressive modification of the contact and the formation of a lithographic track along the sliding path. This behaviour is consistent with localised deformation and limited energy dissipation within the graphene layer (as previously analysed; see Figure 2), leading to stress concentration in the SiO_2_ substrate as sliding proceeds. The amplitude of the stick–slip oscillations remains unaltered throughout the sliding, indicating the existence of sustained and simultaneous interfacial shear resistance as well as a separate mechanism of energy dissipation due to deformation of the SiO_2_ substrate.

In contrast, MoS_2_ exhibits more gradually increasing lateral forces (shown in the lower panel of Figure 3) up to a displacement of 5 nm, for comparable normal loads (i.e., matching of blue and red curves in the two panels of Figure 3). This behaviour suggests elastic energy accumulation in the MoS_2_ layer via deformation induced by the probe’s shear displacement. Such deformation mechanisms include puckering, as illustrated in Figure 1a and quantified in Figure 2a, as well as intralayer deformation. These mechanisms help to postpone the onset of significant interfacial damage. As a result, once the system reaches the yield stress, the MoS_2_ is slit apart, and the underlying substrate begins to deform plastically, though less than graphene under a similar normal load. Regarding stick–slip, we observe a difference between the two normal forces once the MoS_2_ layer ruptures. While for 0.5 µN the stick–slip sawtooth profile remains unaltered throughout sliding, at a higher load of 0.65 µN the sawtooth profile is increasingly smoother, due to an accumulating amount of damage in MoS_2_ as we will demonstrate later in this section.

The effective friction coefficient μ was estimated as the ratio of the mean lateral force $\langle F_{l}\rangle$ and the applied normal load $F_{N}$:

(1) $$\mu =\frac{\langle F_{l}\rangle }{F_{N}}.$$

From Figure 3, we can estimate that the corresponding average lateral forces are higher, 7.6 ± 0.6 and 10.3 ± 0.7 nN for normal loads of 0.5 and 0.65 μN, respectively, in the case of MoS_2_, resulting in an effective friction coefficient of:

(2) $$\mu_{{\mathrm{MoS}}_{2}}\approx 0.015.$$

On the other hand, we can analogously estimate that graphene exhibits average lateral forces 6 ± 1.1 and 9 ± 1.3 nN for normal loads of 0.5 and 0.75 μN, yielding an effective friction coefficient of:

(3) $$\mu_{\mathrm{Gr}}\approx 0.012.$$

From Figure 3, we can see that the elastic energy (estimated as $\Delta F_{l}\Delta x/2$) stored in the MoS_2_-SiO_2_ system before the shear stress levels off is about three times larger than in the graphene-SiO_2_. Approximately, the values are 5 nm·7.6 nN ≈ 38$\cdot 10^{-18}$ J in MoS_2_-SiO_2_ compared to 2 nm·6 nN ≈ 12$\cdot 10^{-18}$ J for graphene-SiO_2_. In this way, MoS_2_ serves as a protective coating that absorbs energy from lateral deformation. Graphene exhibits a much more localised deformation response. Due to its atomic thickness and high in-plane stiffness, graphene resists out-of-plane bending and stores substantially less deformation energy at the contact. The absence of extended puckering is reflected in a per-atom elastic energy of graphene that is 20 times lower than in the case of MoS_2_, as presented in Figure 2b,d.

**Figure 4:**
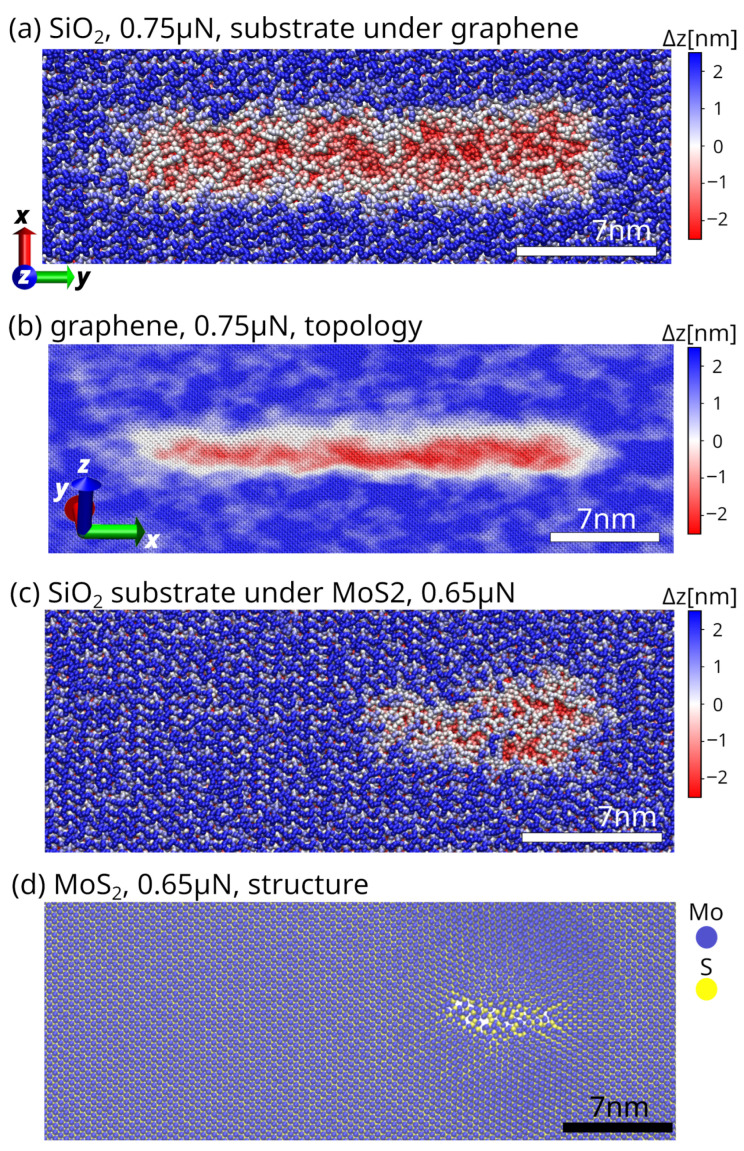
Configuration snapshots illustrating the formation and evolution of lithography tracks formed over the course of nanoscale sliding of the spherical SiO_2_ indenter. In all MD simulations, the sliding direction is along the longer *y*-axis. Panel (**a**): structure of the SiO_2_ substrate under graphene at the applied normal load of 0.75 μN in the initial stage of sliding. Panel (**b**): topology of graphene at the applied normal load of 0.75 μN in the initial stage of sliding. Panel (**c**): structure of the SiO_2_ substrate under MoS_2_ at the applied normal load of 0.65 μN at the end of the sliding path. Panel (**d**): structure of MoS_2_ at the applied normal load of 0.65 μN at the end of the sliding path.

Figure 4 illustrates the formation and evolution of lithography tracks generated during nanoscale sliding contact. The tracks are visualised using both atomistic snapshots and coarse-grained representations, highlighting regions of enhanced plastic deformation and atom removal.

In the initial stage of sliding, i.e., top panels of Figure 4 referring to (a) SiO_2_ substrate under graphene and (b) graphene, a continuous lithography track develops along the sliding direction. This track is characterised by an elongated band of high deformation and energy accumulation spanning several nanometers. The track width exceeds the immediate contact area, indicating that deformation is not confined to a single atomic row, but is redistributed laterally across the 2D layer and the underlying substrate.

The coarse-grained representation emphasises the spatial continuity of the lithography track, revealing a relatively uniform deformation band along the sliding path. This behaviour reflects repeated stick–slip events due to the sliding over the periodic structure of a 2D material, which progressively modifies the underlying substrate surface and accumulates deformation along the indenter’s sliding trajectory. The persistence of the track demonstrates that the material response is dominated by cumulative plastic deformation rather than isolated, reversible stick–slip elastic events, which are confined to the investigated 2D materials.

At the end of the sliding path on MoS_2_, cf. panel of Figure 4c, there is a compact damage zone. This zone is about 5–7 nm long in MoS_2_ and about 3 nm long in the SiO_2_ substrate. The damage region corresponds to the termination of the lithography track, where stresses concentrate, and the probe breaks into a MoS_2_ pucker. Such localisation is indicative of incipient irreversible 2D material deformation, i.e., fracture, material removal, or defect clustering, depending on the local stress state and material properties. The atomic-scale view of fractured MoS_2_ in Figure 4d reveals the microscopic consequence of the prolonged lithography track and accumulation of stress beyond the yield point in pucker, resulting in permanent damage.

In contrast to MoS_2_, in graphene local lattice distortions are observed within the track region, while the surrounding lattice remains largely intact, cf. Figure 2c and Figure 4b. The localisation of graphene deformation under the probe confirms that lithography is not inhibited by highly localised structure rearrangements and defect generation, confined to a narrow region defined by the sliding contact. Overall, configuration snapshots presented in Figure 4 demonstrate that the formation of lithography tracks is prevented by the accumulation of localised deformation in a 2D layer along the sliding path in the form of a pucker.

**Figure 5:**
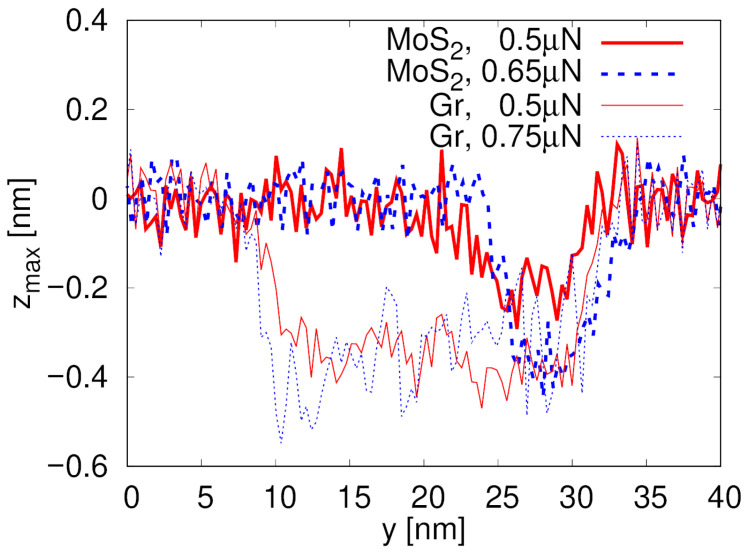
Longitudinal profile of $z_{\max}\left(y\right)$ of the track in SiO_2_ substrate (i.e., along the sliding direction *y*) for MoS_2_ and graphene (Gr) under different normal loads. The quantity $z_{\max}\left(y\right)$ represents the maximum out-of-plane coordinate (along the *z* direction) of SiO_2_ substrate atoms within a slice of width ±0.5 nm centered at position *y*, evaluated at x=0.

**Figure 6:**
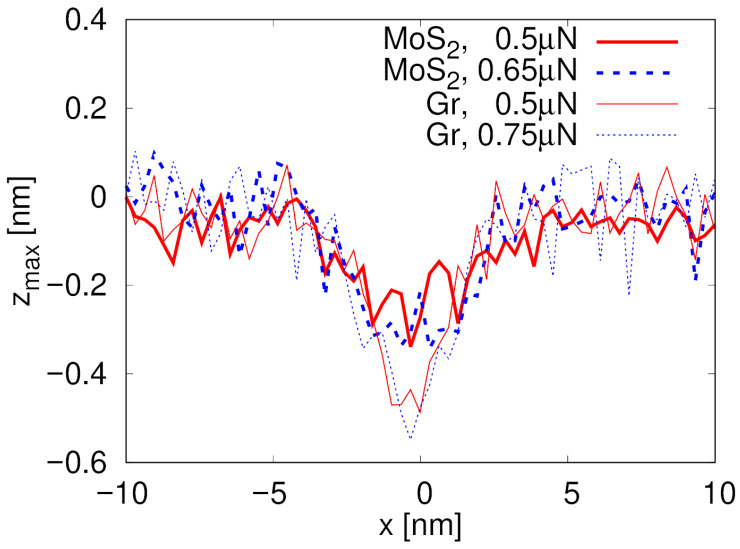
Profile $z_{\max}\left(x\right)$ of a cut through lithographic track in SiO_2_ substrate for MoS_2_ and graphene (Gr) under different normal loads. The quantity $z_{\max}\left(x\right)$ represents the maximum out-of-plane coordinate (along the *z* direction) of SiO_2_ substrate atoms within a slice of width ±0.5 nm centered at position *x*, evaluated at y=28 nm.

Formation and morphological characteristics of the lithography tracks in the SiO_2_ substrate depicted in Figure 4 are quantitatively investigated based on the longitudinal profiles of the track $z_{\max}\left(y\right)$ presented in Figure 5 for both MoS_2_ and graphene (Gr). Here, $z_{\max}\left(y\right)$ denotes the maximum out-of-plane coordinate (along the *z* direction) of SiO_2_ substrate atoms within a slice of width ±0.5 nm centered at position *y*. MoS_2_ exhibits a track shorter than the probe path (about 5 nm out of 25 nm) and approximately 0.19 nm deep the lower simulated normal load, i.e., 0.5 µN see Table 1. It appears only when the material is broken, following the rupture of a pucker beneath the contact as a consequence of the accumulation of lateral strain in the layer, as illustrated in Figure 1a. On the contrary, the $z_{\max}\left(y\right)$ profile for graphene reveals the development of a 22 nm long and 0.4 nm deep trace along the total length of the simulated contact between the probe and the 2D layer. At 0.65 µN normal load, after MoS_2_ is completely ripped apart, the track depth is similar in MoS_2_ and graphene. Figure 6 shows the cross-section profile of the track $z_{\max}\left(x\right)$ at y=28 nm (once the MoS_2_ layer is broken).

## 4. Discussion

In this work, simulation parameters were selected to reproduce the essential contact geometry and loading protocol of AFM-based nanotribology experiments, while remaining compatible with atomistic MD time scales. Accordingly, the results are interpreted primarily in a mechanistic and comparative sense rather than as a direct one-to-one reproduction of experimental sliding rates. The present simulations rely on several modelling assumptions that should be considered when interpreting the results. While the sliding velocity (1 m/s) is higher than in typical AFM experiments (reflecting limitations of the standard molecular dynamics time scale), it is comparable to that in many technical friction systems. The indenter is modelled as a rigid SiO_2_ sphere, with probe deformation neglected. The 2D materials are represented as finite flakes rather than continuous layers to avoid artificial interactions of deformation fields, particularly MoS_2_ puckers, with their periodic images. Stability on the substrate is maintained solely through van der Waals adhesion, similar to that of single-layer nanoflakes of a solid lubricant. Results presented in the Section 3 reveal clear and fundamental differences in the mechanical response, frictional behaviour, as well as wear mechanisms of MoS_2_ and graphene under a nanoscale sliding contact with the SiO_2_ indenter. Although both materials belong to the class of two-dimensional crystals, their distinct out-of-plane responses to the applied load and sliding, along with different internal deformation pathways, lead to fundamentally different nanotribological behaviour. The central finding of this work is the manner in which mechanical energy is accommodated at the contact in the two studied 2D materials—MoS_2_ and graphene.

The per-atom energy distributions demonstrate that MoS_2_ concentrates nearly twenty times more deformation energy within the coating than graphene, cf. Figure 2. This energy localisation is accompanied by the pronounced puckering beneath the contact, as evidenced by both the displacement maps and the $z_{\max}$ profiles. The pucker represents a stable out-of-plane deformation mode that allows MoS_2_ to absorb mechanical energy through bending and interlayer shear, thereby acting as an effective mechanical buffer. As a consequence, a significant fraction of the applied load is taken over by the MoS_2_ layer itself, thus reducing stress transmission to the underlying SiO_2_ substrate. This localised response and low elastic energy absorption result in higher stress concentrations beneath the graphene in SiO_2_, thereby promoting bond distortion and plastic deformation along the entire sliding path. Consequently, graphene accommodates the applied load, leaving a lithographic mark even when the probe is removed.

These substantial differences in deformation mechanisms directly manifest in the lateral force response under comparable normal loads. Both materials exhibit stick–slip motion, characteristic of nanoscale friction in crystalline materials; however, MoS_2_ consistently shows higher average lateral forces and larger stick–slip amplitudes than graphene, at similar normal loads. The rapid levelling off of the mean lateral force with sliding distance for graphene occurs within the first 2 nm. After the initial wear-in process, a 0.4 nm deep wear track is formed. It should be emphasised that direct quantitative comparisons between materials are restricted to the common load of 0.5 µN. At higher loads, the systems operate in different regimes relative to their mechanical stability limits, and the observed differences reflect intrinsic material-dependent failure mechanisms rather than equivalent loading conditions.

In contrast, the stronger dependence of lateral force on displacement for MoS_2_ reflects the gradual formation of a pucker and initially absent wear. Estimated effective friction coefficients further support this reasoning, with MoS_2_ showing higher friction than graphene, only saturating when a rupture point of MoS_2_ under the probe is reached.

The morphology of the lithography tracks provides additional insight into the wear mitigation processes. In MoS_2_, the lithography tracks are absent on the SiO_2_ substrate, consistent with pucker-mediated buffering and delayed severe wear, until the 2D sheet structure is ruptured. In graphene, however, the tracks are characterised by plastically deformed regions and lithographic deformation of the graphene sheet along the track. This behaviour is consistent with the strong localisation of deformation observed in both the out-of-plane displacement and per-atom potential energy analyses. Experimental studies on CVD-grown films have shown that under sufficiently high contact pressures, both graphene and MoS_2_ can undergo rapid removal, with MoS_2_ exhibiting layer-by-layer wear and graphene detaching early due to weaker interfacial bonding [14]. In contrast, the present simulations probe an intermediate loading regime, where graphene remains largely intact while MoS_2_ exhibits localised deformation and rupture, highlighting the load-dependent transition between distinct wear mechanisms. Furthermore, in MoS_2_, additional layers facilitate interlayer sliding, likely increasing resistance to rupture. While the present work focuses on monolayers to isolate fundamental mechanisms, extension to multilayer systems represents a natural and important direction for future study.

Importantly, the correlation between lithography tracks and out-of-plane deformation in 2D materials provides a unifying framework for understanding the nanoscale mechanism for wear prevention. 2D materials capable of forming extended out-of-plane deformation structures, such as puckers in MoS_2_, can effectively accommodate externally applied mechanical disturbances, thereby dissipating energy internally and protecting the underlying substrate. On the contrary, 2D materials with more rigid in-plane bonds, such as graphene, allow deformation and damage only along the sliding trajectory, marginally increasing the radius of an AFM probe or an asperity in the tribological system with dry lubricants such as graphite, leading to increased friction and more pronounced wear.

## 5. Conclusions

All-atom molecular dynamics simulations were used to compare nanoscale friction, lithography, and wear mechanisms in MoS_2_ and graphene under sliding contact. Despite both being two-dimensional materials, their tribological responses differ fundamentally due to their distinct deformation modes. Graphene, with its high in-plane stiffness, exhibits localised deformation and promotes stress concentration in the substrate, leading to plastic damage along the sliding path. In contrast, MoS_2_ accommodates the applied load through out-of-plane puckering, enabling effective energy dissipation, higher friction levels, and delayed wear while protecting the substrate.

These findings highlight the critical role of deformation mechanisms in governing nanoscale friction and wear. Materials capable of redistributing stresses through out-of-plane deformation are particularly effective for tribological applications. While the present results focus on monolayers, the underlying physical mechanisms are expected to remain relevant for multilayer systems, where interlayer effects may further influence the response.

Future work should aim to quantitatively compare friction, failure thresholds, and wear-track formation using matched AFM experiments.

## Figures and Tables

**Table 1:** Summary of key quantities extracted from simulations. Mean lateral force $\langle F_{l}\rangle$ is computed over the steady-state sliding regime after the initial transient, i.e., final 5 nm of the track for graphene and 2 nm for MoS_2_. The track depth is defined as the vertical distance from the reference level given by the z-coordinate of the topmost substrate atoms along the track. The track width and length are measured at half of this depth.

	$F_{N}$	$\langle F_{l}\rangle$	μ	Depth	Length	Width
Material	[µN]	[nN]		[nm]	[nm]	[nm]
Graphene	0.75	9 ± 1.3	0.012	0.35	22.8	3.7 ± 0.4
Graphene	0.5	6 ± 1.1	0.012	0.33	21	3.3 ± 0.2
MoS_2_	0.65	10.3 ± 0.7	0.015	0.29 *	7.1	4.6 ± 0.4
MoS_2_	0.5	7.6 ± 0.6	0.015	0.19 *	7	4.77 ± 0.5

* once MoS_2_ layer is broken.

## Data Availability

The original contributions presented in this study are included in the article. Further inquiries can be directed to the corresponding author.
